# Lung ultrasound combined with C-reactive protein for identifying a bacterial component in children hospitalized with acute lower respiratory tract infections: a prospective observational study

**DOI:** 10.1007/s00431-026-07095-y

**Published:** 2026-06-03

**Authors:** Jiří Fremuth, Tereza Fremuthová, Michal Huml, Eva Sládková, Veronika Schwarzová, Josef Sýkora, Zuzana Rosolová, Jan Forejt, Stanislav Kormunda, Jana Amlerová, Kateřina Chudějová, Jindra Windrichová, Renata Vondráková, Jan Baxa, Martin Pešta, Ondřej Topolčan, Daniel Rajdl

**Affiliations:** 1 Departments of Pediatrics, Faculty Hospital, Faculty of Medicine in Pilsen, Charles University, Pilsen, Czech Republic; 2 Department of Microbiology, Faculty Hospital, Faculty of Medicine in Pilsen, Charles University, Pilsen, Czech Republic; 3 Central Laboratory of Immunoanalysis, Faculty Hospital, Faculty of Medicine in Pilsen, Charles University, Pilsen, Czech Republic; 4 Institute of Clinical Biochemistry and Haematology, Faculty Hospital, Faculty of Medicine in Pilsen, Charles University, Pilsen, Czech Republic; 5 Department of Imaging Methods, Faculty Hospital, Faculty of Medicine in Pilsen, Charles University, Pilsen, Czech Republic; 6 Institute of Biology, Faculty of Medicine in Pilsen, Charles University, Pilsen, Czech Republic

**Keywords:** Lung ultrasound, Chest X-ray, Acute lower respiratory tract infection, Pneumonia, Children, C-reactive protein

## Abstract

Acute lower respiratory tract infections (ALRTIs) remain a major cause of pediatric morbidity and antibiotic use. Early identification of a bacterial component is crucial for rational treatment decisions. We evaluated the diagnostic performance of lung ultrasound (LUS), alone and combined with C-reactive protein (CRP), for identifying a clinically adjudicated bacterial component in children hospitalized with ALRTIs. In this prospective study, 160 children (1 month–19 years) hospitalized with ALRTIs underwent LUS within 24 h of admission. LUS-based presumptive classification (viral, bacterial, combined) was assigned using predefined criteria. Final etiological classification was determined independently by three senior pediatricians blinded to LUS findings, using an adjudicated composite reference standard integrating clinical course, laboratory markers, microbiology, and chest radiography. No prespecified biomarker thresholds were applied. Diagnostic performance for identifying a bacterial component (bacterial or combined vs. viral) was assessed using ROC analysis. Seventy-five children (47%) were classified as viral, 60 (37%) as combined, and 25 (16%) as bacterial/atypical according to the adjudicated composite reference standard. Large consolidations (≥ 20 mm), bronchograms, confluent B-lines, and pleural effusions were associated with a bacterial component, whereas viral cases showed small bilateral subpleural consolidations and non-confluent B-lines. LUS demonstrated good diagnostic accuracy (AUROC 0.89, 95% CI 0.84–0.94; sensitivity 83.5%; specificity 94.6%). CRP ≥ 40 mg/L showed comparable performance (AUROC 0.91, 95% CI 0.87–0.96; sensitivity 75.3%; specificity 94.7%).

*Conclusion*: In children hospitalized with ALRTIs, LUS—particularly alongside CRP—may support early bedside identification of a clinically adjudicated bacterial component and assist treatment decisions.

**Table:** 

**What is Known**: • *Clinical exams and chest X-rays (CXR) poorly distinguish bacterial and combined from viral ALRTIs*. • *Lung ultrasound (LUS) is validated for diagnosing pneumonia, but its role in identifying the etiology is less studied*.	
**What is New**: • *LUS may help identify the presence of a bacterial component in children hospitalized with ALRTIs*. • *CRP was the laboratory marker most strongly associated with bacterial component, and its combination with LUS may support early bedside etiological assessment and more rational antibiotic decision-making*.

**Supplementary Information:**

The online version contains supplementary material available at 10.1007/s00431-026-07095-y.

## Introduction

Acute lower respiratory tract infections (ALRTIs) represent a major global health burden in children. Conditions such as pneumonia, bronchitis, and bronchiolitis remain leading causes of childhood morbidity and mortality, particularly among children under five years of age. ALRTIs are caused by various pathogens, predominantly viruses and bacteria. Pneumococcal pneumonia is the leading bacterial cause of ALRTIs and associated mortality in children [1]. The introduction of childhood vaccination programs against *Streptococcus pneumoniae* and *Haemophilus influenzae type b* has markedly changed the etiology and distribution of pneumonia. Over the past decade, viral infections have played an increasingly dominant role in the etiology of pediatric ALRTIs [1–3].

Timely differentiation and identification of bacterial or atypical etiologies of ALRTIs from non-bacterial infections are essential to guide appropriate therapy and limit antibiotic overuse, particularly in the context of antimicrobial resistance (AMR). AMR remains one of the most serious global health threats. In high-income countries, the antibiotic prescribing rate for acutely ill children in ambulatory care is estimated at around 45%, with approximately one-fifth to one-half of prescriptions considered inappropriate [4, 5]. Recent studies indicate that distinguishing bacterial pneumonia from other causes of pediatric ALRTIs based solely on clinical presentation is challenging [6]. Although combining clinical signs and symptoms may modestly improve diagnostic accuracy, it remains insufficient for reliable differentiation [7–9]. Beyond purely viral or bacterial infections, many children present with combined etiologies (bacterial/atypical plus viral), which are linked to worse outcomes in both children and adults [10]. Therefore, early identification and management of bacterial co-infections are crucial [11, 12].

CXR was long regarded as the reference standard for diagnosing community-acquired pneumonia (CAP) in children with ALRTIs. Current guidelines, however, recommend against the routine use of CXR for diagnostic confirmation of CAP in outpatient settings, restricting its use to hospitalized children with moderate to severe disease [13, 14]. The main reason for this recommendation is the lack of evidence that CXR improves clinical outcomes. Despite the declining role of CXR in both emergency and inpatient settings, most children treated for CAP still undergo this examination [15].

The subjective evaluation of CXR and the differing interpretation of what is required from CXR result in significant interobserver variability in the interpretation of these for CAP [16, 17]. Moreover, CXR does not reliably distinguish between bacterial and viral etiologies of CAP and therefore has limited value in guiding antibiotic decisions [18]. Radiologists continue to refine imaging criteria, standardize interpretive terminology, and assess alternative modalities to improve diagnostic accuracy [17]. LUS has been increasingly used over the past decade to accurately characterize different ALRTIs, monitor disease progression and treatment response, and identify complications [19]. Evidence from both pediatric and adult populations demonstrates that LUS is a reliable tool for diagnosing CAP [19–21]. Nonetheless, despite growing evidence on its diagnostic utility, limited data are available on the ability of LUS to differentiate bacterial from non-bacterial etiologies of ALRTIs in both adults and children. To address this gap, we conducted a single-center prospective observational study. The study aimed to (1) evaluate the diagnostic performance of LUS in identifying the presence of a bacterial component in pediatric ALRTIs; (2) examine how LUS findings relate to CXR findings across adjudicated etiological groups and to explore the potential positioning of LUS within the diagnostic pathway; and (3) develop a practical diagnostic algorithm combining LUS findings and laboratory markers to assist clinicians in accurate etiological classification and antibiotic stewardship.

## Material and methods

This study included children aged 1 month to 19 years with symptoms of ALRTI lasting over 24 h. Patients were enrolled between December 2022 and April 2024 upon admission to the pediatric department of a tertiary referral center. Diagnosis of ALRTI (including pneumonia, bronchiolitis, and bronchitis) was made by emergency physicians based on current guidelines [13, 14, 22]. Inclusion criteria were signs of acute respiratory illness (e.g., cough, fever > 38 °C, tachypnea, dyspnea, chest pain, abnormal auscultation, or oxygen requirement). Although CXR was performed at the physician’s discretion in routine clinical practice, its performance was required for study inclusion. Patients already on antibiotics prior to admission and those transferred from other facilities were also included. Exclusion criteria were (1) chronic respiratory disease or anomalies, (2) immunodeficiency, (3) cystic fibrosis, (4) chronic interstitial lung disease, (5) malignancy, and (6) serious comorbidity preventing imaging. Chronic respiratory disease was defined as conditions associated with structural or functional pulmonary abnormalities that could interfere with LUS interpretation, including bronchopulmonary dysplasia, chronic interstitial lung disease, and other conditions associated with persistent parenchymal changes or fibrosis. These conditions were excluded due to the potential presence of chronic consolidations or fibrotic changes that may mimic acute inflammatory findings on LUS and confound etiological classification. LUS was performed within 24 h of admission by a physician blinded to the clinical, laboratory, and radiologic findings. Based on LUS, patients were categorized into presumed bacterial, viral, or combined etiology groups.

### Etiological classification (composite adjudicated reference standard)

To approximate real-world diagnostic decision-making at hospital admission, the final etiological classification was established using an adjudicated composite reference standard. Three senior pediatricians (each with more than 20 years of clinical experience—SE, SV, HM) independently reviewed all available data and assigned etiology while remaining blinded to the LUS results. The classification framework was informed by previous pediatric studies and international standardization initiatives [23–25]. The adjudication incorporated (i) clinical presentation and disease evolution (including response to treatment), (ii) routine laboratory parameters including inflammatory markers, (iii) microbiological results, and (iv) chest radiography findings. Importantly, adjudicators were not provided with prespecified decision rules or biomarker cut-offs (including CRP) for defining bacterial versus viral infection; all variables were weighed in their overall clinical context. Microbiological confirmation from lower respiratory tract samples was available only in a small subset of patients (e.g., tracheal aspirate, bronchoalveolar lavage, or pleural fluid culture). Consequently, a substantial proportion of cases were classified as probable rather than microbiologically confirmed etiologies, reflecting routine pediatric clinical practice. In all children, nasopharyngeal and/or oropharyngeal samples underwent culture and multiplex PCR testing for a broad panel of viral and bacterial pathogens. These upper respiratory tract findings were considered supportive evidence and were interpreted cautiously due to the possibility of colonization or asymptomatic carriage; they were not regarded as definitive proof of lower respiratory tract causality in the absence of compatible clinical and radiographic features.

Etiology was assigned by majority agreement; any discordance was resolved by consensus discussion. Patients were categorized as follows:

- *Bacterial/atypical (Group B)*: microbiologically confirmed bacterial/atypical infection from a sterile site or lower respiratory tract sample, or a probable bacterial phenotype characterized by compatible clinical presentation together with radiographic consolidation and/or pleural effusion and a marked inflammatory response.
- *Viral (Group V)*: virologically confirmed infection and/or a probable viral phenotype characterized by a compatible clinical course, low or mildly elevated inflammatory markers, and absence of evidence suggesting a bacterial component.
- *Combined (Group C)*: evidence supporting viral infection coexisting with evidence supporting a bacterial/atypical component (probable or confirmed), consistent with bacterial superinfection on a viral background.

This adjudicated composite classification served as the reference standard for evaluating the ability of LUS and laboratory markers to identify the presence of a bacterial component (Group B + C versus Group V). Because routine laboratory and radiographic data contributed to the reference standard, some degree of incorporation bias cannot be fully excluded. Therefore, the observed diagnostic performance should be interpreted as agreement with an adjudicated clinical phenotype rather than with a purely microbiological gold standard.

### Microbiological studies

Nasopharyngeal and oropharyngeal swabs were obtained from all included patients. When available, materials from LRT were also collected, such as sputum, bronchoalveolar lavage, tracheal aspirate or pleural effusion. All collected materials underwent classic microbiological cultivation on conventional media, specifically Columbia Agar with Sheep Blood PLUS and Columbia Agar with Chocolated Horse Blood (Thermo Fisher Scientific, USA), with incubation lasting up to 48 h. The cultivation results were evaluated qualitatively. The bacterial strains were identified using matrix-assisted laser desorption/ionization time-of-flight mass spectrometry (Sirius Bruker, USA). Nasopharyngeal swabs were also used for etiology detection using Real-Time PCR. In the bacterial part of detection, a multiplex panel including Mycoplasma pneumoniae, Chlamydophila pneumoniae, Streptococcus pneumoniae, Haemophilus influenzae, Legionella pneumophila, Branhamella catarrhalis, and Staphylococcus aureus (Fast Track Bacterial pneumonia CAP, Fast Track Diagnostics, Luxembourg) was used. Viral etiology was assessed using several multiplex and singleplex PCR panels—(1) multiplex for detection of Influenza A virus (H1N1 and H3N2), Influenza B virus, and Respiratory syncytial virus (RSV) (Vitassay, Spain); (2) multiplex for detection of Parainfluenza viruses 1–4 (PathoFinder, Netherlands); (3) multiplex for detection of Adenovirus, Bocavirus, Rhinovirus, and Enterovirus (PathoFinder, Netherlands); (4) singleplex for detection of SARS-CoV-2 (Diana Biotechnologies, Czechia); and (5) singleplex for detection of Human Metapneumovirus (Clonit, Italy). The DNA/RNA used for PCR reactions mentioned above was isolated by SaMag™ Viral Nucleic Acid Extraction Kit (Sacace, Italy). Indirect ELISA detecting IgM and IgG antibodies from two paired sera was used in case of suspected viral etiology and etiology of Mycoplasma pneumoniae, Chlamydophila pneumoniae.

### Laboratory studies

In accordance with the study protocol, all patients underwent comprehensive laboratory testing upon admission. The following parameters were assessed: C-reactive protein (CRP), procalcitonin (PCT), interleukin-6 (IL-6), Myxovirus resistance protein A (MxA), and a host-response assay (MeMed BV®), together with standard hematological indices and derived ratios (ICIS score, N/L, L/M, P/L ratios).

CRP, PCT, and IL-6 were analyzed immediately after sampling on the cobas® 702 platform (Roche Diagnostics). CRP was measured by particle-enhanced immunoturbidimetry (Tina-quant CRP IV), and PCT and IL-6 by sandwich chemiluminescent immunoassays (Elecsys BRAHMS PCT, Elecsys IL-6). MxA was determined by chemiluminescent immunoassay on the Kleeya analyzer (BioVendor Group), and the MeMed BV® host-response assay was performed on the DiaSorin LIAISON® XL platform according to the manufacturer’s instructions.

### CXR assessment

Every anteroposterior chest radiograph was downloaded in Digital Imaging and Communications in Medicine (DICOM) format and independently reviewed by two board-certified radiologists (VR, BJ). Both readers were blinded to all clinical and laboratory data, except for patient age. Radiologists applied the modified World Health Organization (WHO) Radiology Working Group 2005 pediatric definitions for community-acquired pneumonia (CAP) [26]. For the purposes of this study, radiographic CAP was defined as the presence of consolidation, other infiltrates (interstitial, alveolar, or mixed), and/or pleural effusion. In cases of disagreement, a consensus was reached during an in-person meeting of the study radiologists.

### LUS assessment

LUS examinations were performed using a Samsung HM60 device. To minimize inter-operator variability, all scans were conducted and interpreted by a single experienced pediatric sonographer (FJ) with over 10 years of expertise in LUS. The operator was blinded to all clinical, laboratory, microbiological, and radiological data at the time of examination.

A linear transducer (3–12 MHz) was used for infants and toddlers, while either a linear or curved probe (1–7 MHz) was employed in older children, depending on chest size. A standardized 12-zone scanning protocol was applied, assessing two anterior, two lateral, and two posterior zones on each hemithorax. Anatomical landmarks for zone delineation included the parasternal, anterior axillary, posterior axillary, and paravertebral lines, as well as the nipple line and diaphragmatic margins.

Children were examined in sitting, supine, or lateral decubitus positions according to age and clinical condition; infants were held by a caregiver when necessary. Scanning was performed in both transverse and longitudinal planes, focusing on the pleural line, with depth and focus adjusted to assess deeper parenchymal pathology (e.g., consolidation, abscess). B-mode imaging was used for all assessments, and color Doppler was applied selectively to evaluate tissue perfusion in abnormal lung areas. Basal segments were examined via subdiaphragmatic acoustic windows through the liver or spleen, and supraclavicular scanning was performed bilaterally to assess apical zones. All findings were recorded and archived as cine loops and static images for subsequent analysis.

### LUS: features, scoring, and diagnostic classification

During each examination, specific sonographic features were systematically assessed to support diagnosis and evaluate disease severity. The following parameters were recorded:

1. Pleural effusion: Classified as simple (anechoic, gravity-dependent fluid collections) or complex (with septations, echogenic content, or loculations) and noted if compressing the lung parenchyma or requiring drainage.
2. B-lines and vertical artifacts: Characterized as long, short, spared, or confluent, originating from the pleural line or perilesional areas, and assessed for distribution (unilateral or bilateral). The presence of ≥ 3 B-lines per intercostal space was considered pathological.
3. Consolidations and atelectasis: Subpleural consolidations were measured and categorized as small (< 20 mm) or large (≥ 20 mm), single or multiple, unilateral or bilateral. Dimensions were recorded in three planes, with the largest value used for analysis.
4. Bronchograms: The presence and type (air or fluid) were documented, along with their dynamic behavior during respiration (static or dynamic).
5. LUS scoring system: Lung involvement was quantified using a semi-quantitative widely adopted score applied to 12 lung zones. The most abnormal finding in each zone was scored from 0 to 3: Score 0: Normal pleural line with A-lines and ≤ 2 B-lines; Score 1: ≥ 3 B-lines in one intercostal space; Score 2: Confluent B-lines or small peripheral consolidations (< 10 mm); Score 3: Large consolidations (> 10 mm). The total LUSS (range 0–36) provided a global estimate of lung aeration and pathology.

Based on LUS findings, patients were categorized into three etiological groups: Viral (V-LUS): Generalized, sparse, bilateral B-lines originating from the pleural surface and/or single or multiple small subpleural consolidations (< 20 mm). Bacterial (B-LUS): Large consolidations (≥ 20 mm), white-lung appearance, and/or complex pleural effusion without viral features. Combined (C-LUS): Combined viral and bacterial characteristics, suggesting bacterial superinfection on a viral background.

### Statistical analysis

Statistical analyses were performed by a licensed statistician (KS) using SAS software (SAS Institute Inc., Cary, NC, USA). Continuous variables were expressed as medians with interquartile ranges (IQR) or means ± standard deviation (SD), as appropriate. Group comparisons were made using the Wilcoxon rank-sum or Kruskal–Wallis test for continuous variables and the *χ*^2^ test or Fisher’s exact test for categorical variables. Diagnostic performance of LUS and laboratory parameters was assessed using receiver operating characteristic (ROC) curve analysis. Optimal cut-off values were derived from the Youden index, and sensitivity, specificity, positive and negative predictive values, positive and negative likelihood ratios and odds ratios with 95% confidence intervals were calculated. Interrater reliability for CXR interpretation was evaluated using Cohen’s κ coefficient and classified according to established thresholds. Multivariable logistic regression with stepwise (score-based) selection was used to identify independent predictors of bacterial component and to select relevant laboratory and imaging variables for further modelling. Input variables included demographic data, clinical symptoms, and laboratory biomarkers (including CRP, procalcitonin, and IL-6). These selected predictors were subsequently entered into a classification and regression tree (CART) analysis to construct a clinically interpretable diagnostic model. The CART algorithm automatically determined the root split and recursively identified the most informative discriminator within each resulting subgroup, thereby generating the second and third levels of the decision tree using recursive binary partitioning. Diagnostic performance metrics, including diagnostic odds ratios, were calculated post hoc for individual decision nodes to quantify the strength of each split. To assess the robustness and reproducibility of the etiological CART model, internal validation was performed using repeated random subsampling (five iterations). Five validation datasets were generated by randomly selecting 100 patients from the full cohort of 160 children. For each subsample, a new CART model was constructed using the same predefined set of laboratory and imaging predictors, and the structure and key decision nodes were compared with the original full-sample model. All tests were two-tailed, and *P* values < 0.05 were considered statistically significant. The dataset is available upon reasonable request.

## Results

Between December 2022 and April 2024, a total of 160 children with ALRTIs were enrolled in the study (Appendix 1 —study flow). Of these, 75 children (46.9%) were classified as viral, 60 (37.5%) as combined viral–bacterial, and 25 (15.6%) as bacterial/atypical according to the adjudicated composite reference standard. The median duration of illness prior to admission was 4 days for the entire cohort (*n* = 160) and did not differ between etiological groups. Children in the adjudicated bacterial infection group were older than those in the adjudicated combined and viral infection groups, while children with viral infections were younger than those with combined infections. These differences in age distribution likely reflect known epidemiological patterns of pediatric respiratory infections. Although age may act as a potential confounder, multivariable analysis identified CRP and LUS features as the primary discriminative factors for the presence of a bacterial component. Admission to the intensive care unit (ICU) was more frequent in children with adjudicated bacterial or combined infections compared with those with adjudicated viral infections. The total length of hospital stay was significantly shorter in the viral group. Key demographic and clinical characteristics are summarized in Table 1, while detailed clinical features, auscultatory findings, and information on oxygen therapy are presented in Appendices 2, 3, and 4. Overall, clinical findings were consistent with the adjudicated etiological classification; notably, asymmetry and decreased breath sounds were more frequently observed in children with adjudicated bacterial and combined etiologies, consistent with focal parenchymal involvement.

**Table 1:** Demographic, clinical, and laboratory characteristics stratified by etiology

	**Bacterial (*n* = 25)**	**Combined (*n* = 60)**	**Viral (*n* = 75)**	*p* value
**Girls**	13 (52%)	34 (57%)	29 (39%)	B:C 0.6934
**Boys**	12 (48%)	26 (43%)	46 (61%)	B:V 0.2421
				C:V 0.0372
**Age (years)**	8.2 (4.5;12.5)	3.9 (1.9;7.6)	0.9 (0.3;2.1)	B:C 0.0038
**median (IQR)**				B:V < 0.0001
				C:V < 0.0001
**Length of illness**	4.0 (3.0; 6.0)	4.0 (3.0; 5.0)	3.0 (2.0; 4.0)	B:C 0.7844
**Before admission (days)**				B:V 0.0787
**Median (IQR)**				C:V 0.0510
**Antibiotics before**	6 (24%)	10 (17%)	9(12%)	B:C 0.5436
**Admission**				B:V 0.1945
**No (%)**				C:V 0.4650
**Need ICU**	9 (36%)	21 (35%)	12 (16%)	B:C 0.9300
				B:V 0.0335
				C:V 0.0107
**ICU days**	4.0 (2.0; 8.0)	3.0 (2.0; 9.0)	2.0 (1.0; 4.0)	B:C 0.9458
**Median (IQR)**				B:V 0.1519
				C:V 0.1521
**Length of hospitalization**	7.0 (5.0; 13.0)	7.0 (5.0; 11.0)	5.0 (3.0; 6.0)	B:C 0.8431
**(days)**				B:V 0.0049
**Median (IQR)**				C:V 0.0001
**Comorbidities**	9 (36%)	18 (30%)	22 (29%)	B:C 0.5882
				B:V 0.5325
				C:V 0.9328
**Antibiotics**	24 (96%)	60 (100%)	41 (55%)	B:C 0.2941
				B:V < 0.0001
				C:V < 0.0001
**Treatment (days)**	7.0 (5.0; 10.0)	6.0 (4.0; 11.0)	3.0 (0.0; 5.0)	B:C 0.8167
**Median (IQR)**				B:V < 0.0001
				C:V < 0.0001
**CRP mg/L**	64 (31; 172)	81 (42; 134)	6.0 (1.0;19.0)	B:C 0.8664
**Median (IQR)**				B:V < 0.0001
				C:V < 0.0001

### Laboratory findings

A comprehensive analysis of laboratory findings will be presented separately. In multivariable logistic regression, CRP emerged as the laboratory parameter most strongly associated with the presence of an adjudicated bacterial component in this cohort. CRP levels were significantly higher in children with adjudicated bacterial and combined etiologies compared with those with adjudicated viral infections, while no significant difference was observed between the adjudicated bacterial and combined groups.

In the final model, CRP ≥ 40 mg/L was strongly associated with bacterial/combined etiology (Odds Ratio 54.1; 95% CI: 17.6–166.0; *p* < 0.0001). Other biomarkers were removed during stepwise selection due to lack of independent contribution in the presence of CRP. A CRP cut-off of ≥ 40 mg/L was derived from the present dataset with the primary aim of achieving 95% specificity while maintaining acceptable sensitivity. At this threshold, CRP demonstrated robust diagnostic performance for identifying an adjudicated bacterial component. Detailed measures of CRP diagnostic performance are summarized in Table 2. Results of microbiological testing are presented in separate tables in Appendix 5.

**Table 2:** Diagnostic performance of CRP and LUS of a bacterial component (B + C vs. V)

Parameter	CRP ≥ 40 mg/L	Lung ultrasound (binary)
Sensitivity (%)	75.3	83.5
Specificity (%)	94.7	94.6
Positive predictive value (PPV, %)	94.1	94.6
Negative predictive value (NPV, %)	77.2	83.5
LR + (95% CI)	14.12 (5.40–36.92)	15.66 (6.01–40.83)
LR − (95% CI)	0.26 (0.18–0.38)	0.17 (0.11–0.28)
Diagnostic odds ratio, DOR (95% CI)	54.10 (17.63–166.02)	90.02 (28.25–286.82)
Accuracy (%)	84.4	88.8
AUROC (95% CI)	0.91 (0.87–0.96)	0.89 (0.84–0.94)

### CXR findings

Chest radiography findings across etiological groups are summarized in Table 3. Consolidation and pleural effusion were predominantly observed in patients with adjudicated bacterial and combined etiologies, whereas the majority of children with adjudicated viral infections showed either interstitial infiltrates or no radiographic evidence of pneumonia. Interobserver agreement between the two radiologists was excellent across all evaluated parameters. Concordance for consolidation reached 94.3% (Cohen’s *κ* = 0.87), for other infiltrates 95.6% (*κ* = 0.91), and for pleural effusion 98.1% (*κ* = 0.94), indicating high reliability of radiographic assessment in this cohort.

**Table 3:** Comparison of CXR and LUS characteristics

	**Bacterial (*n* = 25)**	**Combined (*n* = 60)**	**Viral (*n* = 75)**
**CXR consolidation**	17/25 (68%)	30/60^†^ (50%)	2/75 (3%)
**LUS**	8/17 LUS B	1/30 LUS B	2/2 LUS V
	9/17 LUS C	29/30 LUS C	
**CXR other infiltrate**	4/25 (16%)	24/60^†^ (40%)	31/75 (41%)
**LUS**	2/4 LUS B	6/24 LUS V	3/31 LUS C
	2/4 LUS C	18/24 LUS C	28/31 LUS V
**CXR negative**	4/25 (16%)	6/60 (10%)	42/75 (56%)
**LUS**	2/4 LUS B	6/6 LUS V	1/42 LUS C
	2/4 LUS V		41/42 LUS V
**CXR pleural effusion**	9/25 (36%)	18/60 (30%)	1/75 (1%)
**LUS effusion**	9/9 LUS effusion +	18/18 LUS effusion +	1/1 LUS effusion +
	Extra 2 LUS + and CXR -	Extra 4 LUS + and CXR -	Extra 6 LUS + and CXR -

### LUS findings

LUS findings differed across etiological groups and are summarized in Tables 4 and 5. Large consolidations (≥ 20 mm), bronchograms, confluent B-lines, and pleural effusions were predominantly observed in children with adjudicated bacterial and combined etiologies, whereas adjudicated viral infections were characterized by small, bilateral subpleural consolidations (< 20 mm) and diffuse non-confluent B-lines. Overall LUS scores showed substantial overlap between etiological groups, limiting their value for etiological discrimination.

**Table 4:** Summary of LUS findings (large consolidations and bronchograms) stratified by etiology

	**Bacterial (*n* = 25)**	**Combined (*n* = 60)**	**Viral (*n* = 75)**	*p* value
**Bacterial consolidation**	20 (80%)	46 (76%)	9 (12%)	B:C 0.8355
(≥ 20 mm)				B:V < 0.0001
				C:V < 0.0001
**Size of consolidation**	55.0 (45.0; 77.5)	47.5 (35.0; 60.0)	30.0 (25.0; 35.0)	B:C 0.1201
(mm)				B:V 0.0070
Median (IQR)				C:V 0.0174
**Unilateral consolidation**	18 (72%)	35 (58%)	4 (5%)	B:C 0.0703
				B:V < 0.0001
				C:V < 0.0001
**Bilateral consolidation**	2 (8%)	11 (18%)	5 (7%)	B:C 0.3282
				B:V 1.00
				C:V 0.0372
**Multiple consol. ≥ 2**	2 (8%)	14 (23%)	5 (7%)	B:C 0.1326
				B:V 1.0000
				C:V 0.0057
**Bronchogram**	20 (80%)	37 (62%)	15 (20%)	B:C 0.1013
				B:V < 0.0001
				C:V < 0.0001
**Dynamic bronchogram**	10 (40%)	20 (33%)	3 (4%)	B:C 0.5579
				B:V < 0.0001
				C:V < 0.0001
**Static bronchogram**	17 (68%)	27 (45%)	15 (20%)	B:C 0.0532
				B:V < 0.0001
				C:V 0.0018
**Fluid bronchogram**	18 (72%)	32 (53%)	7 (9%)	B:C 0.1111
				B:V < 0.0001
				C:V < 0.0001

**Table 5:** Summary of LUS findings (distribution patterns of small subpleural consolidations, pleural effusions, B lines, and LUSS score) stratified by etiology

	**Bacterial (*n* = 25)**	**Combined (*n* = 60)**	**Viral (*n* = 75)**	*p* value
**Subpleural consolidations**	10 (40%)	27 (45%)	42 (56%)	B:C 0.6718
**(< 20 mm)**				B:V 0.1655
				C:V 0.2039
**Consolidation size** (mm)	5.5 (4.0; 9.0)	7.0 (4.0; 16.0)	7.0 (4.0; 12.0)	B:C 0.8740
Median (IQR)				B:V 0.4374
				C:V 0.2975
**Multiple consolidations**	5 (20%)	15 (25%)	31 (41%)	B:C 0.6205
				B:V 0.0543
				C:V 0.0466
**Bilateral consolidations**	3 (12%)	13 (22%)	29 (39%)	B:C 0.3739
				B:V 0.0133
				C:V 0.0340
**Pleural effusion**	11 (44%)	22 (37%)	7 (9%)	B:C 0.5273
				B:V 0.0003
				C:V 0.0002
**Pleural distance (mm)**	7.0 (4.0; 15.0)	6.5 (5.0; 18.0)	3.0 (3.0; 3.0)	B:C 0.5579
Median (IQR)				B:V 0.0002
				C:V < 0.0001
**Complex effusion**	2(8%)	4 (7%)	0 (0%)	B:C 1.0000
(drainage/septation)				B:V 0.0606
				C:V 0.0369
**B lines**	24 (96%)	60 (100%)	75 (100%)	B:C 0.2941
				B:V 1.00
				C:V 0.5025
**Unilateral**	6 (24%)	2 (3%)	0 (0%)	B:C 0.0022
				B:V < 0.0001
				C:V 0.1957
**Bilateral**	18 (72%)	58 (97%)	75 (100%)	B:C 0.0022
				B:V < 0.0001
				C:V 0.1957
**Spared**	21 (84%)	57 (95%)	75 (100%)	B:C 0.1872
				B:V 0.0032
				C:V 0.0853
**Confluent**	14 (56%)	32 (53%)	11 (15%)	B:C 0.8221
				B:V < 0.0001
				C:V < 0.0001
**Originating from pleura**	21 (84%)	59 (98%)	75 (100%)	B:C 0.0248
				B:V 0.0032
				C:V 0.4444
**Perilesional**	15 (60%)	38 (63%)	34 (45%)	B:C 0.7726
				B:V 0.2039
				C:V 0.0372
**Quadrants with B lines**	7.0 (6.0; 8.0)	12.0 (11.0; 12.0)	12.0(12.0; 12.0)	B:C 0.0034
**Originating from pleura**				B:V < 0.0001
Median (IQR)				C:V < 0.0001
**LUS score**	16.0 (9.0; 18.0)	16.0 (14.0; 17.5)	13.0 (12.0; 16.0)	B:C 0.2350
Median (IQR)				B:V 0.9290
				C:V 0.0004

For the purpose of identifying an adjudicated bacterial component, LUS demonstrated good diagnostic performance. Detailed measures of diagnostic accuracy, including sensitivity, specificity, predictive values, likelihood ratios, diagnostic odds ratio, overall accuracy, and the area under the receiver operating characteristic curve, are summarized in Table 2. A comparative overview of LUS and chest radiography findings across adjudicated etiological groups is provided in Table 3.

### Classification and regression tree (CART)

In the CART analysis, the predefined set of laboratory and imaging variables identified in the primary analysis was used to assess their relative contribution to adjudicated etiological differentiation. Among these variables, CRP and selected LUS features consistently emerged as the most informative predictors of an adjudicated bacterial component. Across the five internal validation runs, three decision trees were fully identical to the original model with respect to structure and variable hierarchy. In the remaining two validation trees, minor differences were observed, confined to secondary splits within the left-sided branches of the tree. Importantly, the right-sided branches—representing the main discriminative pathway—remained identical to the original model in all validation runs. These variations reflected substitution of secondary predictors by closely related ultrasound parameters and were consistent with variability expected from reduced sample size in the validation subsets. No changes affecting the overall decision logic or primary discriminating variables were observed. The overall concordance between the validation trees and the original model was 89.9%, suggesting reasonable internal consistency of the model structure.

The final CART model demonstrates the combined diagnostic contribution of CRP and LUS and provides a transparent, clinically interpretable framework to support early identification of an adjudicated bacterial component. Building directly on the diagnostic performance of CRP and LUS summarized in Table 2, the model integrates the strongest individual predictors into a unified decision pathway. The model is intended to support, not replace, clinical decision-making.

**Fig. 1:**
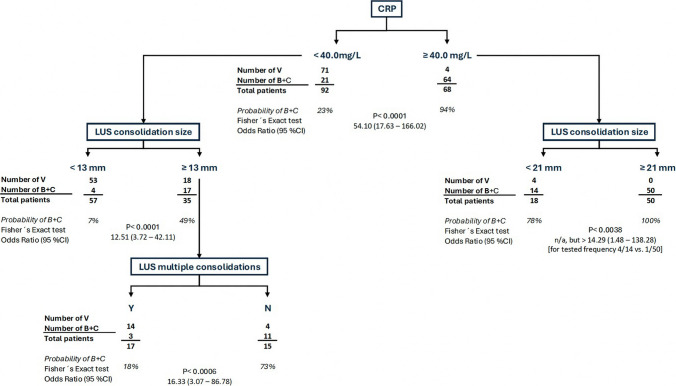
CART model for adjudicated etiological differentiation of ALRTIs. *Legend:* The model integrates all laboratory and imaging parameters, identifying CRP as the root node and key LUS findings—size of consolidations, as the main branching variables distinguishing adjudicated bacterial from non-bacterial infections. The resulting model reflects a clinically relevant decision-making pathway and highlights key LUS variables for diagnostic stratification

The structure of the final decision tree is shown in Fig. 1.

## Discussion

In this prospective study, we evaluated the role of LUS and CRP in identifying a clinically adjudicated bacterial component in pediatric acute lower respiratory tract infections, reflecting a clinically relevant decision point for antibiotic initiation. Our findings demonstrate that LUS provides clinically relevant information aligned with adjudicated etiological classification and complements laboratory biomarkers in early risk stratification. The integration of ultrasound findings with inflammatory markers offers a pragmatic, bedside-oriented approach to support clinical decision-making in children with suspected lower respiratory tract infections.

### Diagnostic role of LUS

Large, predominantly unilateral consolidations (≥ 20 mm), dynamic or fluid bronchograms, and complex pleural effusions were strongly associated with bacterial and combined infections, whereas viral cases typically presented with small, multiple, bilateral subpleural consolidations (< 20 mm) and diffuse, non-confluent B-lines.

These findings align with prior pediatric studies supporting the role of LUS in etiological differentiation. Berce et al. showed that viral pneumonias present with smaller consolidations (median 15 mm) than bacterial pneumonias (median 30 mm), with a 21-mm cutoff yielding 80% sensitivity and 75% specificity for distinguishing bacterial from viral CAP [23]. Our data confirm this pattern and identified a slightly higher optimal threshold (27 mm) with comparable performance (87% sensitivity, 79% specificity), although this cutoff should be considered exploratory pending external validation. Similarly, Buonsenso et al. reported detailed associations between consolidation size, bronchograms, pleural effusions, and vertical artifacts and predefined etiological categories [24]. Notably, these studies primarily evaluated how individual LUS features correlate with established diagnoses. Malla et al. also demonstrated high diagnostic accuracy of LUS in differentiating bacterial from viral pneumonia and pragmatically grouped mixed cases with bacterial pneumonia for therapeutic purposes [27]. However, their comparison relied on a clinicoradiological reference standard in which chest radiography was treated as the gold standard. In contrast, our study prospectively assigned an etiological classification based solely on predefined ultrasound criteria under complete blinding to clinical, laboratory, microbiological, and radiographic information, and benchmarked this against an independent adjudicated composite reference standard. Thus, rather than examining feature-level associations alone, our design evaluates LUS as a stand-alone etiological classifier and specifically addresses the presence of a bacterial component (bacterial or combined vs. viral), directly reflecting the antibiotic decision point at hospital admission. Berce et al. observed that atypical pneumonia resembled viral patterns with small, bilateral consolidations [23]. Buonsenso et al. noted that atypical pneumonia was associated with multiple bilateral subpleural consolidations typically less than 4 cm with dynamic air bronchograms [24]. As only 10 cases of Mycoplasma pneumoniae infection were included in our study, they mainly presented as large, unilateral consolidations rather than diffuse patterns. Although this may reflect sample size and disease spectrum, it aligns with findings by Liu et al. and others, who observed that mycoplasma infections can present as large consolidations, sometimes associated with refractory or severe courses [28, 29].

### LUS scoring and disease severity

Using a standardized 12-zone protocol, we applied a semi-quantitative LUS score to quantify aeration loss. In our cohort, LUS scores showed considerable overlap between etiologies, limiting their usefulness for reliable etiological differentiation. This aligns with findings from recent pediatric literature, which generally supports the role of LUS scoring in evaluating severity and monitoring clinical course rather than identifying the underlying cause [30–33]. A recent Italian study by Morello et al. demonstrated the potential of LUSS to differentiate bacterial from viral pneumonia; however, their data also showed a substantial overlap of values between the two etiologies [34].

### Comparison with chest radiography

Chest radiography remains widely used in the evaluation of pediatric ALRTIs, despite its limitations in etiological discrimination. In a study of over 2000 children with pneumonia, consolidation appeared in 74% of bacterial and 54% of viral cases, but its positive predictive value for bacterial pneumonia was only 12% [18, 35]. However, its absence strongly excluded bacterial CAP (NPV 96%). Contrary to this, in our study consolidation was frequent in bacterial and combined groups but rare in purely viral cases, suggesting that its presence makes viral etiology unlikely. Consistent with prior research, complicated pleural effusion in our LUS and CXR findings was strongly associated with a bacterial component [18, 23, 24, 35]. Previous studies reported only fair interobserver agreement, particularly among non-radiologists, with the lowest agreement observed for subjective findings. Using standardized WHO criteria and independent interpretation by two experienced radiologists, our study demonstrated high interobserver agreement, exceeding that reported in previous studies [36–40]. In our cohort, LUS and CXR showed overlapping but not identical imaging patterns across adjudicated etiological groups. While consolidation and pleural effusion were predominantly associated with an adjudicated bacterial component on both modalities, LUS provided additional bedside morphological detail—particularly regarding consolidation size, bronchogram characteristics, and the distribution and pattern of B-lines—that support early etiological stratification at hospital admission. While the present study was specifically designed to evaluate early etiological assessment at hospital admission, it did not address longitudinal follow-up or monitoring of disease evolution. In current clinical practice, chest radiography is often used to document resolution in selected cases, particularly in patients with complicated or non-resolving pneumonia. However, growing evidence suggests that lung ultrasound may be useful for monitoring disease progression and treatment response, with the potential to reduce exposure to ionizing radiation [41, 42].

### Laboratory markers and CRP

Inflammatory markers complement imaging in differentiating bacterial and non-bacterial infections. In our cohort, CRP outperformed all other biomarkers, including PCT, IL-6, MxA, and the host-response MeMed BV® assay. A CRP threshold of ≥ 40 mg/L yielded 75.3% sensitivity and 94.7% specificity (AUROC 0.91). These results mirror those of the PERCH study, which reported that CRP ≥ 40 mg/L differentiated bacterial from RSV pneumonia with 77% sensitivity and 82% specificity (AUROC 0.87) [43]. Our findings also align with Gunaratnam et al.’s meta-analysis, which reported a lower diagnostic accuracy at a higher threshold (53 mg/L, AUROC 0.71) [44]. Recent evidence suggests that, despite new molecular and host-response assays, CRP remains the most practical and cost-effective biomarker for everyday clinical use [44, 45]. Our findings suggest limited etiological specificity of nasopharyngeal bacterial detection in this context, likely reflecting colonization rather than true LRT infection. In contrast, viral PCR results were more consistent with clinical phenotypes, underscoring the need for cautious interpretation of upper respiratory tract microbiology in children with ALRTIs.

### CART model and diagnostic pathway

A major strength of this study is the integration of imaging and laboratory data into a transparent and clinically interpretable decision model. The CART model illustrates a potential decision pathway based on the most informative LUS features (consolidation size and count) and laboratory parameters identified in this cohort. Rather than serving as a prescriptive tool, the resulting decision tree provides a structured framework that reflects how CRP and selected LUS findings may jointly contribute to etiological assessment at the bedside.

Such integrative approaches may be particularly useful at hospital admission, when decisions regarding antibiotic initiation are frequently required before microbiological results become available [46–48]. In this context, the combination of LUS and CRP may support clinical reasoning, facilitate early risk stratification, and contribute to more rational antibiotic use in pediatric patients.

### Clinical implications

Our findings support the use of LUS as a first-line imaging modality in children hospitalized with ALRTIs, particularly when interpreted in conjunction with CRP. This approach may facilitate early, pathogen-oriented stratification at hospital admission, help reduce unnecessary antibiotic prescribing, and decrease reliance on radiographic imaging. The portability, absence of ionizing radiation, and scalability of LUS make it particularly well suited for high-throughput pediatric units and for settings with limited radiologic or microbiological resources [15, 17, 20]. These characteristics underscore its potential applicability not only in tertiary care centers but also in decentralized and resource-constrained environments.

### Strengths and limitations

This study has several strengths. Its prospective design and inclusion of a well-characterized cohort of hospitalized children with ALRTIs allowed for standardized clinical, laboratory, and imaging assessment. LUS examinations were performed according to a predefined protocol, and etiological classification was based on a composite reference standard incorporating microbiological, clinical, and radiological data reviewed by experienced clinicians blinded to LUS findings. Importantly, the study focuses on the detection of a bacterial component, reflecting a clinically meaningful decision point rather than strict etiological categorization.

The integration of LUS and CRP using CART analysis represents an additional strength, providing a transparent and clinically interpretable framework that illustrates how imaging and laboratory data can be combined to support bedside decision-making.

Several limitations should be acknowledged. This was a single-center study, which may limit generalizability to other clinical settings. The relatively modest sample size limits statistical power and precludes definitive conclusions. Therefore, the present study should be interpreted as hypothesis-generating and exploratory, providing a basis for future multicenter validation. LUS examinations were performed by a single experienced operator, and interobserver variability was therefore not assessed. Although the predefined ultrasound-based etiological classification was applied prospectively under strict blinding, its reproducibility across operators with varying levels of ultrasound expertise remains to be established and warrants external validation. Although the etiological classification was comprehensive, limited availability of lower respiratory tract samples may have constrained microbiological confirmation in some cases. In addition, the relatively small number of atypical infections precluded detailed subgroup analyses. Although a composite reference standard was used to approximate real-world clinical practice, misclassification of etiology cannot be fully excluded. The reference standard reflected an adjudicated clinical phenotype rather than a purely microbiological gold standard; therefore, the reported diagnostic performance represents agreement with clinical classification rather than definitive etiological truth. Because chest radiography and laboratory parameters formed part of the composite reference standard, some degree of incorporation bias cannot be excluded. The 27-mm consolidation threshold was derived from ROC analysis within the present dataset using the Youden index and should therefore be interpreted as exploratory, requiring external validation before clinical implementation. Finally, the CART model was internally validated only and should be considered hypothesis-generating, requiring external validation before broader clinical implementation.

Despite these limitations, the standardized methodology, clinically relevant outcome, and pragmatic integration of imaging and laboratory data strengthen the validity and potential applicability of the findings.

## Conclusion

In children hospitalized with ALRTIs, LUS, particularly when interpreted alongside CRP, may support early bedside identification of a clinically adjudicated bacterial component and contribute to more rational antibiotic decision-making. The integration of imaging and laboratory information underscores the potential role of LUS as a first-line imaging approach in selected pediatric patients with ALRTIs and warrants further evaluation in diverse clinical settings. These findings should be interpreted in the context of a single-center, relatively small cohort and require confirmation in larger, multicenter studies.

## Supplementary Information

Below is the link to the electronic supplementary material.

ESM1 Appendix A (JPG 674 KB)

ESM2 Appendix B. Frequency of selected symptoms and signs stratified by infection etiology (DOCX 15.7 KB)

ESM3 Appendix C. Frequency of auscultatory findings stratified by infection etiology (DOCX 16.2 KB)

ESM4 Appendix D. Oxygen therapy and pleural intervention/treatment stratified by infection etiology (DOCX 16.8 KB)

ESM5 Appendix E. Detailed results of microbiological and virological testing stratified by etiology (DOCX 26.6 KB)

## Data Availability

Collected clinical data, laboratory data, and results from chest X-rays and LUS are available upon request.

## Abbreviations

ALRTI: Acute lower respiratory tract infection
AMR: Antimicrobial resistance
AUROC: Area under the receiver operating characteristic curve
BAL: Bronchoalveolar lavage
B-LUS: Bacterial lung ultrasound pattern
CAP: Community-acquired pneumonia
CART: Classification and regression tree
CI: Confidence interval
CRP: C-reactive protein
CXR: Chest X-ray
C-LUS: Combined lung ultrasound pattern
DICOM: Digital Imaging and Communications in Medicine
ELISA: Enzyme-linked immunosorbent assay
HFNO: High flow nasal oxygenation
ICIS: Inflammatory cell index score
ICU: Intensive care unit
IgG: Immunoglobulin G
IgM: Immunoglobulin M
IL-6: Interleukin-6
IQR: Interquartile range
L/M: Lymphocyte-to-monocyte ratio
LRT: Lower respiratory tract
LUS: Lung ultrasound
LUSS: Lung ultrasound score
MxA: Myxovirus resistance protein A
MV: Mechanical ventilation
NIV: Non-invasive ventilation
N/L: Neutrophil-to-lymphocyte ratio
NPV: Negative predictive value
PCR: Polymerase chain reaction
PCT: Procalcitonin
PICU: Pediatric intensive care unit
PPV: Positive predictive value
ROC: Receiver operating characteristic
RSV: Respiratory syncytial virus
RT-PCR: Real-time polymerase chain reaction
SARS-CoV-2: Severe acute respiratory syndrome coronavirus 2
SD: Standard deviation
V-LUS: Viral lung ultrasound pattern
WHO: World Health Organization
