# Supplementary figures and images for: Lung ultrasound combined with C-reactive protein for identifying a bacterial component in children hospitalized with acute lower respiratory tract infections: a prospective observational study
Source: Eur J Pediatr. 2026 Jun 3;185(6):458. doi: 10.1007/s00431-026-07095-y (PMC13230270; doi:10.1007/s00431-026-07095-y)

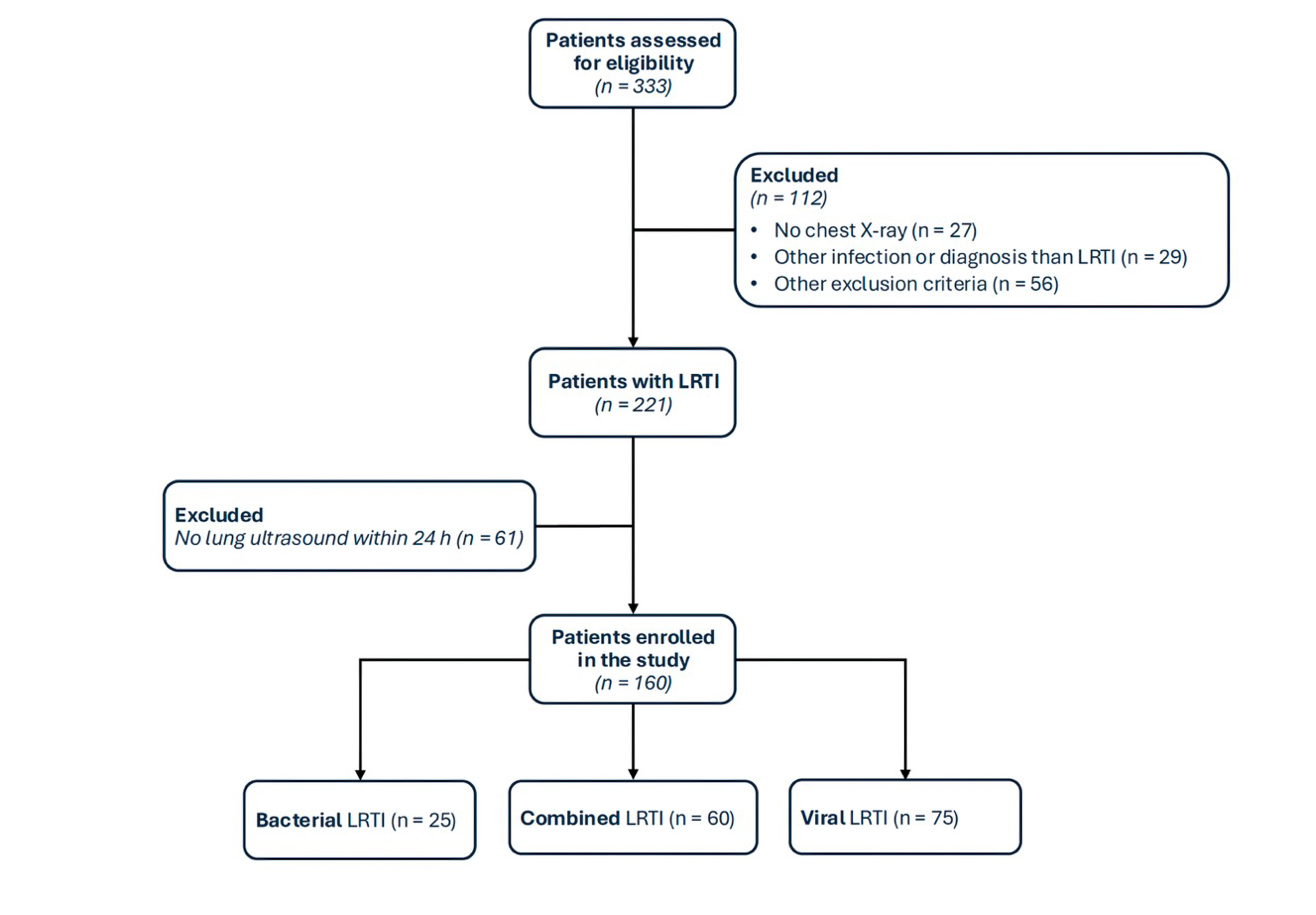

Supplement: Supplementary file 1 — Appendix A (JPG 674 KB) [file 431_2026_7095_MOESM1_ESM.jpg]
