# Supplementary material for: Lung ultrasound combined with C-reactive protein for identifying a bacterial component in children hospitalized with acute lower respiratory tract infections: a prospective observational study
Source: Eur J Pediatr. 2026 Jun 3;185(6):458. doi: 10.1007/s00431-026-07095-y (PMC13230270; doi:10.1007/s00431-026-07095-y)
Supplement: Supplementary file 2 — Appendix B. Frequency of selected symptoms and signs stratified by infection etiology (DOCX 15.7 KB) [file 431_2026_7095_MOESM2_ESM.docx]

Appendix B. Frequency of selected symptoms and signs stratified by infection etiology.

|  | **Bacterial (n=25)** | **Combined (n=60)** | **Viral (n=75)** | p-value |
| --- | --- | --- | --- | --- |
| **Fever** | 23 (92%) | 55 (92%) | 47 (63%) | B:C 1.0000 |
|  |  |  |  | B:V 0.0053 |
|  |  |  |  | C:V <0.0001 |
| **Cough** | 22 (88%) | 59 (98%) | 74 (99%) | B:C 0.0744 |
|  |  |  |  | B:V 0.0472 |
|  |  |  |  | C:V 1.0000 |
| **Increased work** | 5 (32%) | 38 (63%) | 61 (81%) | B:C 0.0083 |
| **of breathing** |  |  |  | B:V <0.0001 |
|  |  |  |  | C:V 0.0188 |
| **Tachypnea** | 11 (44%) | 40 (67%) | 44 (59%) | B:C 0.0519 |
|  |  |  |  | B:V 0.2018 |
|  |  |  |  | C:V 0.3408 |
| **Tachycardia** | 8 (32%) | 25 (42%) | 28 (37%) | B:C 0.4047 |
|  |  |  |  | B:V 0.6304 |
|  |  |  |  | C:V 0.6084 |
