# Supplementary material for: Lung ultrasound combined with C-reactive protein for identifying a bacterial component in children hospitalized with acute lower respiratory tract infections: a prospective observational study
Source: Eur J Pediatr. 2026 Jun 3;185(6):458. doi: 10.1007/s00431-026-07095-y (PMC13230270; doi:10.1007/s00431-026-07095-y)
Supplement: Supplementary file 3 — Appendix C. Frequency of auscultatory findings stratified by infection etiology (DOCX 16.2 KB) [file 431_2026_7095_MOESM3_ESM.docx]

Appendix C. Frequency of auscultatory findings stratified by infection etiology.

|  | **Bacterial (n=25)** | **Combined (n=60)** | **Viral (n=75)** | p-value |
| --- | --- | --- | --- | --- |
| **Asymmetry** | 19 (76%) | 33 (55%) | 8 (11%) | B:C 0.0703 |
|  |  |  |  | B:V <0.0001 |
|  |  |  |  | C:V <0.0001 |
| **Rhonchi** | 2 (8%) | 9 (15%) | 33 (44%) | B:C 0.4952 |
|  |  |  |  | B:V 0.0011 |
|  |  |  |  | C:V 0.0003 |
| **Wheezes** | 0 (0%) | 12 (20%) | 36 (48%) | B:C 0.0152 |
|  |  |  |  | B:V <0.0001 |
|  |  |  |  | C:V 0.0007 |
| **Crackles** | 8 (32%) | 33 (55%) | 40 (53%) | B:C 0.0532 |
|  |  |  |  | B:V 0.0645 |
|  |  |  |  | C:V 0.8469 |
| **Decreased breath sounds** | 17 (68%) | 32 (53%) | 10 (13%) | B:C 0.2124 |
|  |  |  |  | B:V <0.0001 |
|  |  |  |  | C:V <0.0001 |
| **Tubular breathing** | 2 (8%) | 8 (13%) | 3 (4%) | B:C 0.7161 |
|  |  |  |  | B:V 0.5964 |
|  |  |  |  | C:V 0.0613 |
