# Supplementary material for: Lung ultrasound combined with C-reactive protein for identifying a bacterial component in children hospitalized with acute lower respiratory tract infections: a prospective observational study
Source: Eur J Pediatr. 2026 Jun 3;185(6):458. doi: 10.1007/s00431-026-07095-y (PMC13230270; doi:10.1007/s00431-026-07095-y)
Supplement: Supplementary file 4 — Appendix D. Oxygen therapy and pleural intervention/treatment stratified by infection etiology (DOCX 16.8 KB) [file 431_2026_7095_MOESM4_ESM.docx]

Appendix D. Oxygen therapy and pleural intervention/treatment stratified by infection etiology.

|  | **Bacterial (n=25)** | **Combined (n=60)** | **Viral (n=75)** | p-value |
| --- | --- | --- | --- | --- |
| **O2 therapy** | 10 (40%) | 46 (77%) | 59 (79%) |  |
|  |  |  |  |  |
| **Nebulisation** | 6 (24%) | 25 (42%) | 39 (52%) | B:C 0.1231 |
|  |  |  |  | B:V 0.0148 |
|  |  |  |  | C:V 0.2322 |
| **HFNO** | 3 (12%) | 15 (25%) | 20 (27%) | B:C 0.1813 |
|  |  |  |  | B:V 0.1313 |
|  |  |  |  | C:V 0.8262 |
| **NIV** | 0 (0%) | 1 (2%) | 0 (0%) | B:C 1.0000 |
|  |  |  |  | B:V 1,000 |
|  |  |  |  | C:V 0.4444 |
| **MV** | 1 (4%) | 5 (8%) | 0 (0%) | B:C 0.6655 |
|  |  |  |  | B:V 0.2500 |
|  |  |  |  | C:V 0.0158 |
| **MV (days)** | 5(0) | 7.5 (3.70) | 0 (0) | B:C 1.0000 |
| mean (SD) |  |  |  | B:V |
|  |  |  |  | K:V |
| **Pleural drain** | 3 (12%) | 4 (6.67%) | 0 (0%) | B:C 0.4145 |
|  |  |  |  | B:V 0.0142 |
|  |  |  |  | C:V 0.0369 |
| **Fibrinolysis** | 3 (12%) | 3 (5%) | 0 (0%) | B:C 0.3533 |
|  |  |  |  | B:V 0.0142 |
|  |  |  |  | C:V 0.0853 |
