# Supplementary material for: Lung ultrasound combined with C-reactive protein for identifying a bacterial component in children hospitalized with acute lower respiratory tract infections: a prospective observational study
Source: Eur J Pediatr. 2026 Jun 3;185(6):458. doi: 10.1007/s00431-026-07095-y (PMC13230270; doi:10.1007/s00431-026-07095-y)
Supplement: Supplementary file 5 — Appendix E. Detailed results of microbiological and virological testing stratified by etiology (DOCX 26.6 KB) [file 431_2026_7095_MOESM5_ESM.docx]

Appendix E. Detailed results of microbiological and virological testing stratified by etiology.

| **Nasopharyngeal swab culture** | |  |  |  |
| --- | --- | --- | --- | --- |
|  | **Bacterial (n=25)** | **Combined (n=60)** | **Viral (n=75)** | |
| **Streptococcus pneumoniae** | 5 (20%) | 18 (30%) | 10 (13%) | B:C 0.3444 |
|  |  |  |  | B:V 0.5182 |
|  |  |  |  | C:V 0.0176 |
| **Haemophilus influenzae** | 3(12%) | 12 (20%) | 14 (19%) | B:C 0.5362 |
|  |  |  |  | B:V 0.5505 |
|  |  |  |  | C:V 0.8452 |
| **Moraxella catarrhalis** | 2 (8%) | 7 (12%) | 15 (20%) | B:C 1 |
|  |  |  |  | B:V 0.2261 |
|  |  |  |  | C:V 0.1927 |
| **Staphylococcus aureus** | 4 (16%) | 6 (10%) | 11 (15%) | B:C 0.4708 |
|  |  |  |  | B:V 1.0000 |
|  |  |  |  | C:V 0.4167 |
| **Streptococcus pyogenes** | 0 (0%) | 4 (7%) | 0 (0%) | B:C 0.3152 |
|  |  |  |  | B:V |
|  |  |  |  | C:V 0.0369 |
| **Klebsiella pneumoniae** | 0 | 0 | 0 | B:C |
|  |  |  |  | B:V |
|  |  |  |  | C:V |
| **Other pathogens** | 1 (4%) | 4 (7%) | 3 (4%) | B:C 1.0000 |
|  |  |  |  | B:V 1.0000 |
|  |  |  |  | C:V 0.6995 |
| **Multipathogen isolates** |  |  |  |  |
| **0** | 11 (44%) | 16 (27%) | 25 (33%) | B:C 0.1075 |
| **1** | 13 (52%) | 39 (65%) | 46 (61%) | B:V 0.3359 |
| **2** | 2(4%) | 2 (3%) | 4 (5%) | C:V 0.3476 |
| **3** | 0 (0%) | 3 (5%) | 0 (0%) |  |

| **PCR nasopharyngeal swab - bacterial** | |  |  |  |
| --- | --- | --- | --- | --- |
|  | **Bacterial (n=25)** | **Combined (n=60)** | **Viral (n=75)** | |
| **Mycoplasma pneumoniae** | 6 (24%) | 4 (7%) | 0 (0%) | B:C 0.0576 |
|  |  |  |  | B:V 0.0001 |
|  |  |  |  | C:V 0.0369 |
| **Haemophilus influenzae** | 8 (32%) | 29 (48%) | 23 (31%) | B:C 0.1664 |
|  |  |  |  | B:V 0.9007 |
|  |  |  |  | C:V 0.0361 |
| **Streptococcus pneumoniae** | 6 (24%) | 25 (42%) | 16 (21%) | B:C 0.1231 |
|  |  |  |  | B:V 0.7804 |
|  |  |  |  | C:V 0.0107 |
| **Moraxella catarrhalis** | 5 (20%) | 17 (28%) | 31 (41%) | B:C 0.4241 |
|  |  |  |  | B:V 0.0543 |
|  |  |  |  | C:V 0.1169 |
| **Staphylococcus aureus** | 7 (28%) | 13 (22%) | 27 (36%) | B:C 0.5305 |
|  |  |  |  | B:V 0.4646 |
|  |  |  |  | C:V 0.0699 |
| **PCR multipathogen isolates** | |  |  |  |
| **0** | 7 (28%) | 12 (20%) | 15 (20%) | B:C 0.3248 |
| **1** | 8 (32%) | 17 (28%) | 31 (41%) | B:V 0.8923 |
| **2** | 6 (24%) | 22 (37%) | 23 (31%) | C:V 0.1590 |
| **3** | 4 (16%) | 9 (15%) | 4 (5%) |  |
| **4** | 0(0%) | 0 (0%) | 2 (3%) |  |

**PCR nasopharyngeal swab - viral**

|  | **Bacterial (n=25)** | **Combined (n=60)** | **Viral (n=75)** |  |
| --- | --- | --- | --- | --- |
| **RSV** | 0 (0%) | 8 (13%) | 28 (37%) | B:C 0.0981 |
|  |  |  |  | B:V 0.0003 |
|  |  |  |  | C:V 0.0017 |
| **Influenza** | 0 (0%) | 11 (18%) | 7 (9%) | B:C 0.0290 |
|  |  |  |  | B:V 0.1874 |
|  |  |  |  | C:V 0.1264 |
| **Adenovirus** | 0 (0%) | 4 (7%) | 3 (4%) | B:C 0.3152 |
|  |  |  |  | B:V 0.5710 |
|  |  |  |  | C:V 0.6995 |
| **Rhino/enterovirus** | 0 (0%) | 20 (33%) | 23 (31%) | B:C 0.0010 |
|  |  |  |  | B:V 0.0016 |
|  |  |  |  | C:V 0.7411 |
| **Bocavirus** | 0 (0%) | 4 (7%) | 4 (5%) | B:C 0.3152 |
|  |  |  |  | B:V 0.5695 |
|  |  |  |  | C:V 1.0000 |
| **Parainfluenza** | 0 (0%) | 7 (12%) | 4 (5%) | B:C 0.0995 |
|  |  |  |  | B:V 0.5695 |
|  |  |  |  | C:V 0.2159 |
| **Metapneumovirus** | 0 (0%) | 8 (13%) | 6 (8%) | B:C 0.0981 |
|  |  |  |  | B:V 0.3321 |
|  |  |  |  | C:V 0.3125 |
| **COVID 19** | 0 (0%) | 2 (3%) | 4 (5%) | B:C 1.0000 |
|  |  |  |  | B:V 1.0000 |
|  |  |  |  | C:V 0.6924 |
| **PCR multipathogen isolates** | |  |  |  |
| **0** | 24 (96%) | 9 (15%) | 11 (15%) | B:C <0.0001 |
| **1** | 0 (0%) | 40 (67%) | 50 (67%) | B:V <0.0001 |
| **2** | 0 (0%) | 9 (15%) | 13 (17%) | C:V 0.9473 |
| **3** | 0 (0%) | 2 (3%) | 1 (1%) |  |

| **Blood culture** | **Bacterial (n=25)** | **Combined (n=60)** | **Viral (n=75)** |  |
| --- | --- | --- | --- | --- |
| **Positive** | 1(4%) | 3 (5%) | 0 (0%) | B:C 1.0000 |
|  |  |  |  | B:V 0.2500 |
|  |  |  |  | C:V 0.0853 |
| **Performed** | 8 (32%) | 9 (15%) | 7 (9%) |  |
|  |  |  |  |  |

| **Pneumococcal antigen in urine** | **Bacterial (n=25)** | **Combined (n=60)** | **Viral (n=75)** |  |
| --- | --- | --- | --- | --- |
| **Positive** | 2 (8%) | 15 (25%) | 6 (8%) | B:C 0.0742 |
| **Negative** | 21 (84%) | 35 (58%) | 33 (44%) | B:V 1.0000 |
| **Not performed** | 2 (8%) | 10 (17%) | 36 (48%) | C:V 0.0068 |
|  |  |  |  |  |

| **Broncho-Alveolar lavage culture** | **Bacterial (n=25)** | **Combined (n=60)** | **Viral (n=75)** |  |
| --- | --- | --- | --- | --- |
| **Performed** | 2(8%) | 4 (7%) | 0 (0%) | B:C 1.0000 |
| **Positive** | 2(8%) | 4 (7%) | 0 (0%) | B:V 0.0606 |
| **Not performed** | 23 (92%) | 56 (93%) | 75 (100%) | C:V 0.0369 |
